# MicroRNA-137 reduces stemness features of pancreatic cancer cells by targeting KLF12

**DOI:** 10.1186/s13046-019-1105-3

**Published:** 2019-03-12

**Authors:** Zhiwei He, Xingjun Guo, She Tian, Changhao Zhu, Shiyu Chen, Chao Yu, Jianxin Jiang, Chengyi Sun

**Affiliations:** 1 Guizhou Medical University, Guiyang, China; 2 Department of Hepatic-Biliary-Pancreatic Surgery, The Affiliated Hospital of Guizhou Medical University, Guiyang, China; 3 Key Laboratory of Hepatobiliary and Pancreatic Surgery, Guiyang, China; 4 Department of Biliary-Pancreatic Surgery, Affiliated Tongji Hospital, Tongji Medical College, Huazhong University of Science and Technology, Wuhan, China; 5 Department of Hepatic-Biliary-Pancreatic Surgery, Renmin Hospital of Wuhan University, Wuhan, China; 6 Hubei Key Laboratory of Digestive System Disease, Wuhan, China

**Keywords:** miR-137, Pancreatic cancer, KLF12, Stemness

## Abstract

**Background:**

Cancer stem cells (CSCs) play an important role in the development of pancreatic cancer. We previously showed that the microRNA miR-137 is downregulated in clinical samples of pancreatic cancer, and its expression negatively regulates the proliferation and invasiveness of pancreatic cancer cells.

**Methods:**

The stemness features of pancreatic cancer cells was detected by flow cytometry, immunofluorescence and sphere formation assay. Xenograft mouse models were used to assess the role of miR-137 in stemness features of pancreatic cancer cells in vivo. Dual-luciferase reporter assays were used to determine how miR-137 regulates KLF12. Bioinformatics and Chromatin immunoprecipitation analysis of KLF12 recruitment to the DVL2 promoters. Involvement of the Wnt/β-catenin pathways was investigated by western blot and Immunohistochemistry.

**Results:**

miR-137 inhibits pancreatic cancer cell stemness in vitro and vivo. KLF12 as miR-137 target inhibits CSC phenotype in pancreatic cancer cells. Suppression of KLF12 by miR-137 inhibits Wnt/β-catenin signalling. KLF12 expression correlates with DVL2 and canonical Wnt pathway in clinical pancreatic cancer.

**Conclusion:**

Our results suggest that miR-137 reduces stemness features of pancreatic cancer cells by Targeting KLF12-associated Wnt/β-catenin pathways and may identify new diagnostic and therapeutic targets in pancreatic cancer.

**Electronic supplementary material:**

The online version of this article (10.1186/s13046-019-1105-3) contains supplementary material, which is available to authorized users.

## Background

Pancreatic cancer is one of the most aggressive and lethal malignancies worldwide [1]. Despite effective surgical resection and systemic chemo-radiotherapy, the overall survival rate for pancreatic cancer remains very low [2]. Cancer stem cells (CSCs) represent a small subpopulation of cells within tumors; however, they have the ability to self-renew, play vital roles in tumor initiation and metastasis in multiple cancers, and are typically chemo-radiotherapy resistant [3]. In pancreatic tumors, CSCs account for 0.2–0.8% of the total cancer cell population and are regarded as vital for malignant behavior [4, 5]. Furthermore, a specific subpopulation of cells that contains CSC characteristics has been identified in human pancreatic cancers [6]. However, despite recent advances related to CSC isolation and characterization, the mechanisms that regulate CSC formation in pancreatic tumors remain largely unknown.

Various oncogenes and signaling pathways contribute to CSC maintenance and tumorigenicity. These include BMI1 (B-cell-specific Moloney murine leukemia virus insertion site 1), LGR5 (leucine-rich repeat-containing G-protein-coupled receptor 5), NANOG (Nanog homeobox), OCT4A (POU class 5 homeobox 1), and SOX2 (SRY-box 2), [7]. These pluripotency genes have important functions during embryonic development and participate in the maintenance of CSC phenotypes by directly targeting genes that control tumor stemness, survival, proliferation, invasion, and chemo-radiotherapy resistance [3, 8]. Transcripts of the above genes exert control over multiple morphogenetic signaling pathways, including Wnt/β-Catenin TGFβ, and Hedgehog, that are aberrantly active in many human tumors [9, 10]. β-catenin is the main effector of the Wnt pathway. It directly binds to TCF/LEF transcription factors to upregulate the expression of downstream genes, promoting cell proliferation, metastasis, and chemo-resistance [11]. β-catenin is phosphorylated by GSK3β within a ‘destruction complex’ formed with APC and AXIN1, resulting in β-catenin ubiquitination and degradation [12]. Therefore, suppressing β-catenin phosphorylation prevents formation of the destruction complex and β-catenin degradation. Non-phosphorylated β-catenin is translocated to the nucleus and interacts with TCF/LEF to regulate downstream gene expression [13]. Activation of β-catenin may increase the CSC population in pancreatic cancer, while inhibition of the Wnt/β-catenin pathway blocks the tumor-promoting activity of pancreatic CSCs [14]. When the Wnt/β-catenin pathway is activated, these receptors phosphorylate the cytoplasmic protein Dishevelled (Dvl), which protects β-catenin from a protein complex containing APC, GSK3, Axin. This promotes β-catenin nuclear translocation, as well as transcription of Wnt/signaling target genes [15–17]. Thus, exploring new ways to target Wnt/β-catenin might lead to novel and successful treatments for pancreatic cancer.

MicroRNAs (miRNAs) are short non-coding RNAs, 17–25 nucleotides long, that regulate target genes post-transcriptionally [18]. Multiple miRNAs have been shown to control CSC maintenance and tumorigenicity [19]. Among these miRNAs, It was reported that miR-137 exhibited both oncogenic and tumor-suppressive roles in the context of different cancer types. For example, Chang TH al. found that miR-137 is a Slug-induced miRNA that relays the pro-metastatic effects of Slug by targeting TFAP2C in in non-small cell lung cancer (NSCLC) [20]. miR-137 was also reported to play a tumor suppressor in various tumor. miR-137 can regulate XIAP via its 3′UTR sensitise ovarian cancer cells to cisplatin-induced apoptosis in ovarian cancer [21]. Our previous studies showed that the miR-137 inhibits pancreatic cancer cell proliferation, invasion and chemoresistance through targeting PTN in pancreatic cancer [22]. Recently, Neault M found that miR-137 inhibits the bypass of Ras-induced senescence and triggers the p53 and p16INK4A tumor suppressor pathways by targeting KDM4A in pancreatic cancer [23]. However, the mechanism of miR-137 in pancreatic cancer stem cells has not been fully elucidated. Therefore, it is of great significance to reveal the molecular mechanism of abnormal expression of miR-137 for understanding the pathogenesis of pancreatic cancer. In this study, We firstly demonstrated that KLF12 was a direct target of miR-137, and down-expression of miR-137 significantly promoted stemness by down-regulating KLF12 via activating Wnt/β-catenin pathway. Hence, our study identified a new molecular signaling pathway for understanding the pathogenesis and of pancreatic cancer.

## Materials and methods

### Chemicals and cell culture

All the antibodies were purchased from Cell Signaling Technology, Inc. (Danvers, MA, USA) unless otherwise noted. The human pancreatic cancer cell lines AsPC-1 and PANC-1 were obtained from American Type Culture Collection (ATCC, Manassas, VA, USA) and cultured at 37 °C in a humidified 5% CO_2_ incubator according to ATCC protocols. PANC-1 cells were grown in DMEM medium and AsPC-1 cells were grown in RPMI-1640 medium (Gibco, NY, USA). Culture media were supplemented with 10% fetal bovine serum (Gibco), 100 U/mL penicillin G, and 100 μg/ml streptomycin (Sigma, MO, United States).

### RNA preparation and real-time PCR

Tissue harvesting and processing and PCR were conducted according to standard procedures. RNA was reverse-transcribed to cDNA using a qPCR RT Kit (Takara, Japan). Quantitative real-time PCR was performed using the SYBR Green Realtime PCR Premix (Takara, Japan), according to the manufacturer’s instructions. qRT-PCR primers as Supplementary Material 1:Table 1.

**Table 1:** The characteristics of the primers used for real-time PCR

GENE	Sequence (5′ - > 3′)Sequence (5′ - > 3′)	
BMI-1	Forward Primer	CCACCTGATGTGTGTGCTTTG
	Reverse Primer	TTCAGTAGTGGTCTGGTCTTGT
ABCG2	Forward Primer	CAGGTGGAGGCAAATCTTCGT
	Reverse Primer	ACCCTGTTAATCCGTTCGTTTT
NANOG	Forward Primer	TTTGTGGGCCTGAAGAAAACT
	Reverse Primer	AGGGCTGTCCTGAATAAGCAG
SOX2	Forward Primer	GCCGAGTGGAAACTTTTGTCG
	Reverse Primer	GGCAGCGTGTACTTATCCTTCT
OCT4A	Forward Primer	CTGGGTTGATCCTCGGACCT
	Reverse Primer	CCATCGGAGTTGCTCTCCA
LGR5	Forward Primer	CTCCCAGGTCTGGTGTGTTG
	Reverse Primer	GAGGTCTAGGTAGGAGGTGAAG
KLF12	Forward Primer	CGGCAGTCAGAGTCAAAACAG
	Reverse Primer	CGGCTTCCATATCGGGATAGT
TCF4	Forward Primer	CAAGCACTGCCGACTACAATA
	Reverse Primer	CCAGGCTGATTCATCCCACTG
CCND1	Forward Primer	GCTGCGAAGTGGAAACCATC
	Reverse Primer	CCTCCTTCTGCACACATTTGAA
MYC	Forward Primer	GGCTCCTGGCAAAAGGTCA
	Reverse Primer	CTGCGTAGTTGTGCTGATGT
TWIST1	Forward Primer	GTCCGCAGTCTTACGAGGAG
	Reverse Primer	GCTTGAGGGTCTGAATCTTGCT
CD44	Forward Primer	CTGCCGCTTTGCAGGTGTA
	Reverse Primer	CATTGTGGGCAAGGTGCTATT
MMP7	Forward Primer	GAGTGAGCTACAGTGGGAACA
	Reverse Primer	CTATGACGCGGGAGTTTAACAT
Control	UUAUUGCUUAAGAAUACGCGUAG	
	UCACAACCUCCUAGAAAGAGUAGA	
inhibitor	CTACGCGTATTCTTAAGCAATAA	
miR-137 mimic	UUAUUGCUUAAGAAUACGCGUAG	

### Generation of stable cell lines

Human miR-137 overexpressing (miR-137), knockdown (anti-miR-137), and negative-control (NC) lentiviruses were purchased from Genechem (Shanghai, China). All transfections were carried out according to the manufacturer’s instructions.

### Plasmids, siRNAs, and transfection

Cells were seeded in 6-well plates at 50% confluence without antibiotics on the day before transfection with siRNAs (RiboBio, Guangzhou, China). For luciferase reporter assays, pairs of oligonucleotides containing the 3′-UTR binding site for miR-137 (RiboBio, Guangzhou, China) were used. Transfection of miRNAs, siRNAs, and plasmids was performed using Lipofectamine 3000 (Life Technologies Co., Carlsbad, CA, USA) according to the manufacturer’s instructions. The siRNA is used at a concentration of 50 nM and the transfection time is 48 h.The sequence of miR-137-control: 5′-UCACAACCUCCUAGAAAGAGUAGA-3′, sequence of miR-137-mimic: 5′-UUAUUGCUUAAGAAUACGCGUAG-3′, sequence of miR-137-5′-inhibitor: 5′-CTACGCGTATTCTTAAGCAATAA-3′. sequence of small-interfering RNA sequence of KLF12: 5′-GCAATCGAATGAATAATCA-3′.

### Sphere formation assay

Cells (500/well) were seeded into 6-well ultra-low attachment cluster plates (Corning, NY, USA) and cultured in serum-free DMEM/F12 medium (Invitrogen, Carlsbad, CA, USA) supplemented with 2% B27 (Invitrogen), 20 ng/ml EGF (PeproTech), 20 ng/ml bFGF (PeproTech), 0.4% BSA (Sigma-Aldrich), and 5 μg/ml insulin (Sigma-Aldrich). After two weeks, spheres were photographed and counted.

### Flow cytometric analysis

Cells were dissociated with trypsin, resuspended at 1 × 10^6^ cells per ml in 200 μl DMEM containing 2% FBS and subsequently incubated with PE-conjugated CD133 antibody (MiltenyiBiotec, Germany) for 90 min at 37 °C. Cells were then incubated on ice for 10 min and washed with ice-cold PBS before flow cytometric analysis. Isotype-matched immunoglobulins served as controls.

### Tumor xenografts

All experimental procedures were approved by the Institutional Animal Care and Use Committee (IACUC) of Guizhou Medical University. Six-week-old BALB/c-nu mice were randomly divided into eight groups (*n* = 4 per group), and implanted with 1 × 10^6^, 1 × 10^5^, 1 × 10^4^ or 1 × 10^3^ cells previously transfected with negative control or miR-137-overexpressing lentiviruses. Cells were mixed with Matrigel (50% volume) and implanted subcutaneously into the inguinal folds of the nude mice. Tumor volume was determined using an external caliper and calculated using the eq. (L × W^2^)/2. Mice were sacrificed 35 days after inoculation. At this point, tumors were excised and subjected to downstream analyses.

### Subcellular fractionation assay

Subcellular fraction was obtained from cultured cells and tissues using Subcellular Protein Fractionation Kit for Cultured Cells and Subcellular Protein Fractionation Kit for Tissues (Thermo Fisher Scientific).

### Immunofluorescence

Cells grown on glass plates were fixed with 4% paraformaldehyde. Next, the cells were incubated with the primary antibody (1:100) at 4 °C overnight followed by Alexa Fluor 594-conjugated goat anti-rabbit IgG (1:250). Slides were counterstained with DAPI to visualize cell nuclei. Images were recorded using a confocal laser scanning microscope.

### Histology and immunostaining

Immunohistochemistry assays were performed on the paraffin-embedded Subcutaneous implants tumor and pancreatic cancer tissue, using the primary antibodies as Supplementary materials. The degree of immunostaining of indicated proteins was evaluated and scored by two independent observers, who scored both the proportions of tumor cells that stained positively and the intensity of the staining.

### Statistical analysis

All values are presented as means ± standard deviation (SD). Statistical analyses were performed using SPSS 22.0 software (SPSS, Chicago, USA). Student’s t-test was used to determine statistical differences. *P* < 0.05 was considered significant.

## Results

### miR-137 inhibits pancreatic cancer cell stemness in vitro

**Fig. 1:**
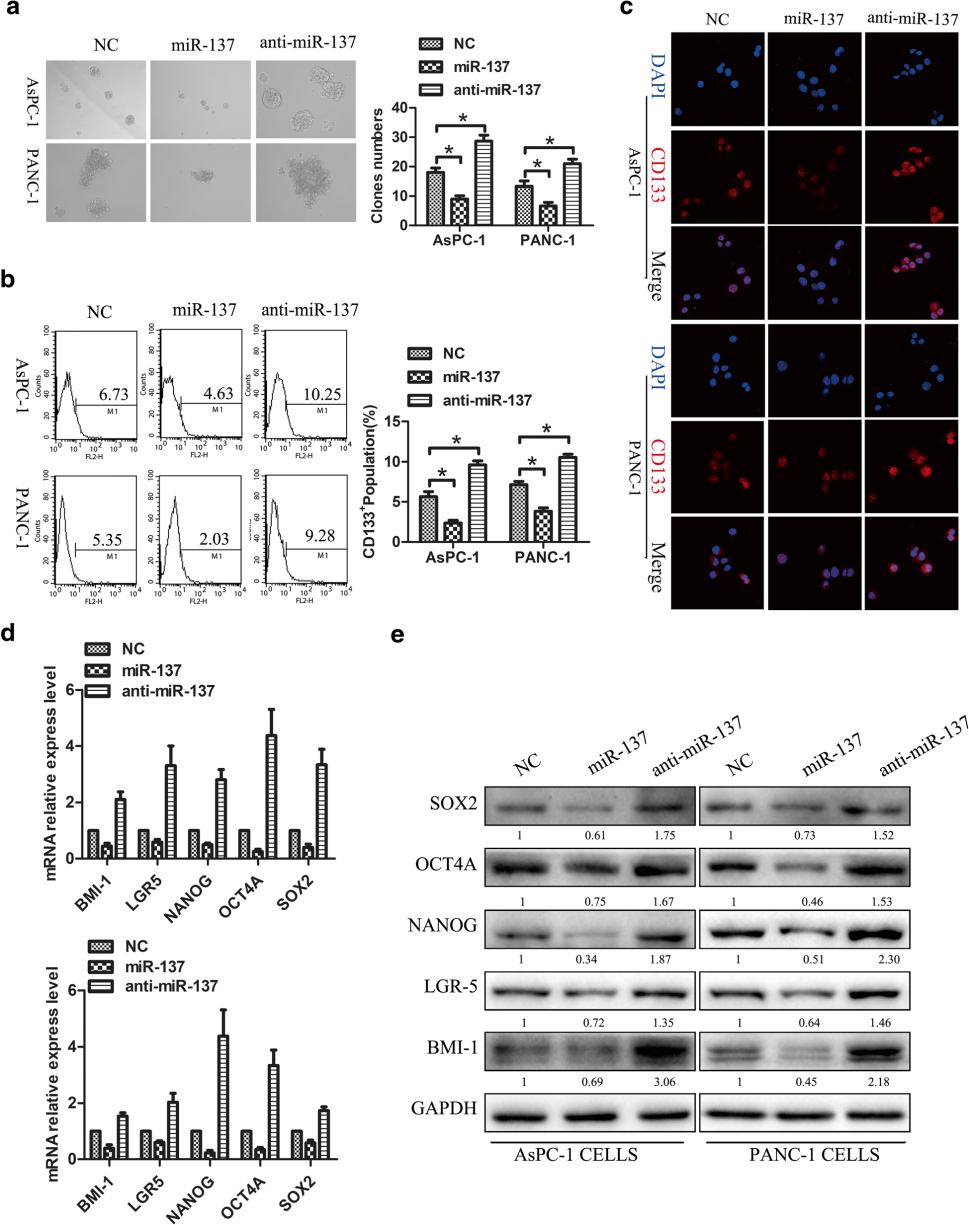
miR-137 inhibits pancreatic cancer cell stemness in vitro. **a** miR-137 upregulation dramatically decreased the size and number of spheres formed in AsPC-1 and PANC-1 cell cultures, while anti-miR-137 had the opposite effect (*p* < 0.05). **b**-**c** Overexpression of miR-137 decreased the CD133^+^ population in cultured AsPC-1 and PANC-1 cells. In contrast, anti-miR-137 increased the CD133^+^ population in both cell lines (*p* < 0.05).**d-e** Overexpression of miR-137 markedly downregulated the expression of pluripotency-related mRNAs and proteins (*p* < 0.05), whereas miR-137 knockdown significantly upregulated the expression of these markers (*p* < 0.05)

We reported that miR-137 is markedly downregulated in pancreatic cancer, contributing to tumor growth, invasion, and resistance to chemotherapy. To determine whether miR-137 levels affect the self-renewal of pancreatic CSCs, the human pancreatic cancer cell lines PANC-1 and AsPC-1 were transfected with miR-137 mimic (miR-137) or inhibitor (anti-miR-137) in vitro. As shown in Fig. 1a, upregulation of miR-137 dramatically decreased the size and number of spheres in both cell lines, while anti-miR-137 stimulated sphere formation. Since CD133 expression has been reported to characterize CSC-like populations in many tumors, including pancreatic cancer, the effect of miR-137 on the CD133^+^ populations of AsPC-1 and PANC-1 cells was examined by flow cytometry and immunofluorescence. Overexpression of miR-137 decreased CD133^+^ cell populations in both cell lines, while miR-137 downregulation had the opposite effect in Fig. 1b-c. These results indicate that miR-137 inhibits the CSC phenotype in human pancreatic cancer cells in vitro. Several transcription factors, including BMI1, LGR5, NANOG, OCT4, and SOX2, are upregulated in various cancer types and promote stemness maintenance, local tumor invasion, and distant metastasis. To assess whether alterations in miR-137 expression affect the expression of these markers, RT-PCR and Western-blot were conducted in AsPC-1 and PANC-1 cells treated with miR-137 mimics or inhibitors. As expected, overexpression of miR-137 significantly downregulated the expression of these genes both at the mRNA and protein levels, whereas miR-137 inhibition had the opposite effect in Fig. 1d-e. These results suggest that miR-137 inhibits CSC reprogramming by repressing the transcription of several pluripotency-related genes.

### miR-137 inhibits pancreatic cancer cell tumorigenesis in vivo

**Fig. 2:**
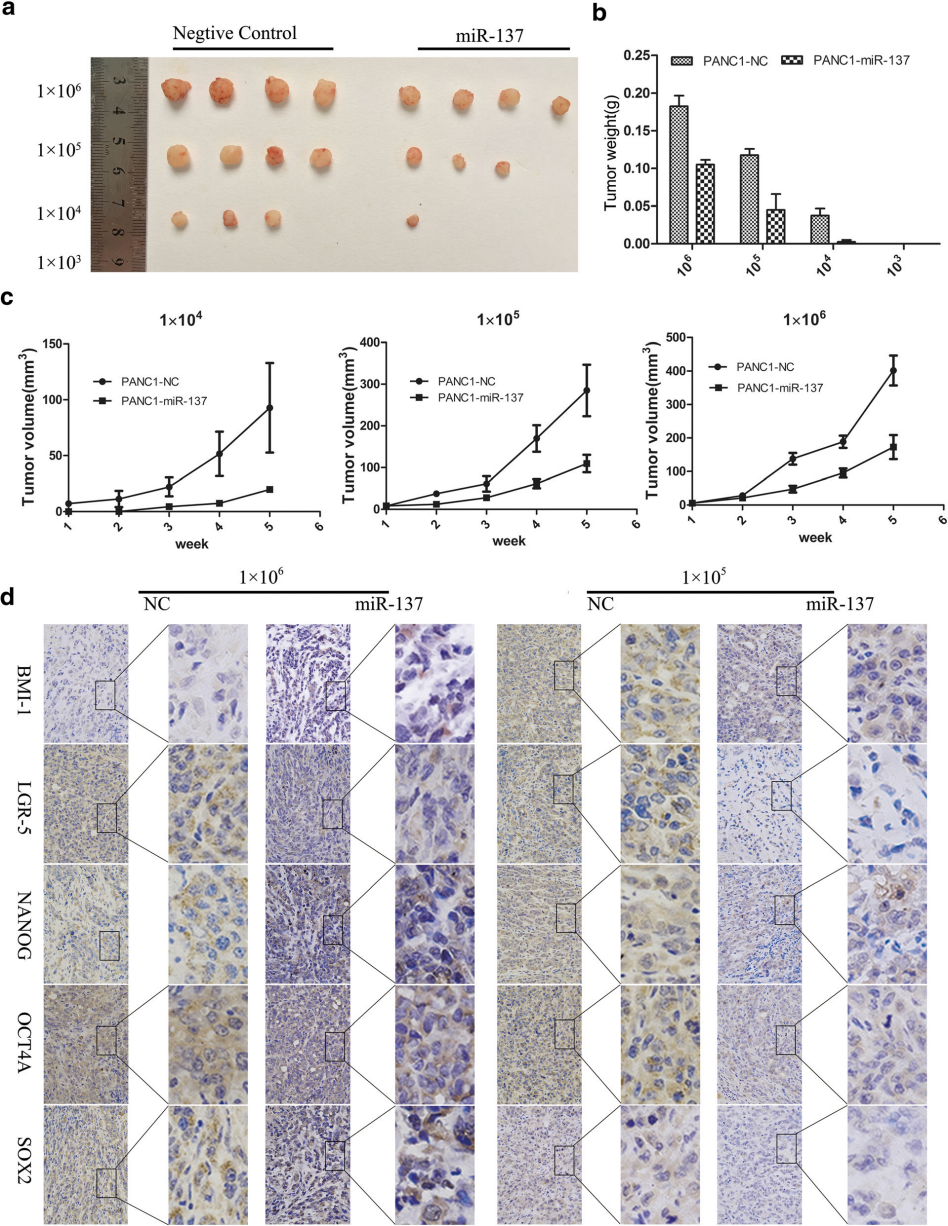
miR-137 inhibits pancreatic cancer cell tumorigenesis in vivo. **a** Images of the subcutaneous xenografts from the miR-137-overexpressing and control groups. **b** Tumors weight were significantly lower in the miR-137-overexpressing group compared with the control group. **c** Tumors in the miR-137-overexpressing group grew more slowly than tumors in the control group. **d** Immunohistochemical detection of pluripotency-associated markers in excised tumors

To test the involvement of miR-137 on human pancreatic cancer cells’ tumorigenesis in vivo, PANC-1 cells transfected with miR-137 overexpression or negative control lentivirus were implanted into nude mice at different seeding densities (1 × 10^6^, 1 × 10^5^, 1 × 10^4^ or 1 × 10^3^ cells). As shown in Fig. 2a-c, miR-137-overexpressing tumors grew more slowly than tumors in the negative control group. Moreover, whereas no tumors developed in either group after implantation of 1 × 10^3^ cells, only negative control-transfected cells formed tumors when 1 × 10^4^ cells were implanted. Ex-vivo tumor immunohistochemistry analyses revealed significantly higher levels of pluripotency-associated markers in negative control specimens compared with miR-137-overexpressing tumors in Fig. 2c. These results demonstrate that miR-137 reduces CSC marker expression and inhibits pancreatic cancer cell tumorigenesis in vivo.

### KLF12 as miR-137 target inhibits CSC phenotype in pancreatic cancer cells

**Fig. 3:**
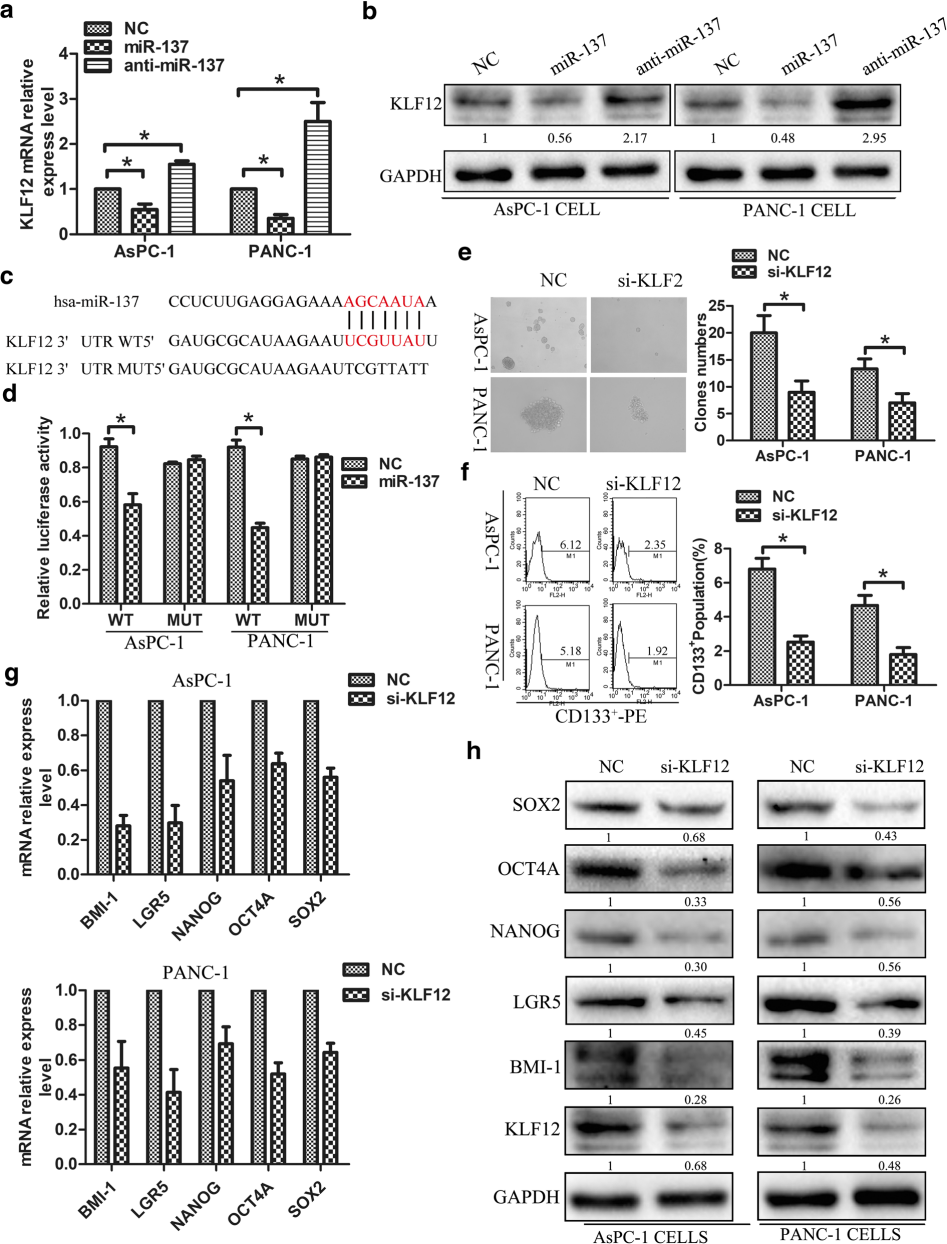
KLF12 as miR-137 target inhibits CSC phenotype in pancreatic cancer cells. **a**-**b** PCR and western blot analysis the KLF12 expression in miR-137-mimic and miR-137-inhibitor transfected AsPC-1 and PANC-1 cells. **c** Predicted binding sites of miR-137 in the wild type 3-UTRs of KLF12,Mutations in these 3-UTRs are highlighted in red. **d** Luciferase activity of reporters with wild type or mutant 3-UTR of KLF12 in the indicated cells co-transfected with the indicated oligonucleotides **e** *KLF12* knockdown significantly impaired tumor-sphere formation in both in AsPC-1 and PANC-1 cells. **f** KLF12 knockdown decreased the CD133^+^ population in AsPC-1 and PANC-1 cells **g** Real-time PCR and western blot analyses showed that downregulation of KLF12 inhibited the expression of pluripotency-associated markers in AsPC-1 and PANC-1 cells

To explore potential targets through which miR-137 inhibits the CSC phenotype in pancreatic cancer cells, we searched publicly available algorithms (miRanda and TargetScan) and identified KLF12. Figure 3a-b revealed that KLF12 expression in AsPC-1 and PANC-1 cells was decreased after miR-137 upregulating,but was increased after miR-137 downregulating. To examine whether direct miR-137-mediated KLF12 downregulation occurs through a miR-137-binding site in KLF12 3′-UTR, the latter sequence was cloned into the pGL3 dual luciferase reporter vector. Results showed that miR-137 overexpression effectively reduced luciferase reporter activity of the KLF12 3′-UTR. In contrast, the activity of the KLF12 3′-UTR luciferase reporter containing point mutations (mut) in the miR-137-binding seed region was unaffected by miR-137 overexpression in Fig. 3c-d. These results indicate that miR-137 directly targets KLF12.To analyze the role of KLF12 in CSC formation and proliferation, PANC-1 and AsPC-1 cell lines were transfected with KLF12 siRNA. As expected, KLF12-knockdown significantly impaired the ability to form tumor spheres in Fig. 3e, and also decreased CD133^+^ populations in both cell lines in Fig. 3f. Furthermore, the expression of stemness markers, as assessed by RT-PCR and western blotting, was significantly downregulated by KLF12 knockdown in Fig. 3.g-h. These results suggest that miR-137 directly targets KLF12 and KLF12 activity contributes to the CSC phenotype in human pancreatic cancer cells.

### KLF12 mediates CSC phenotype induction after miR-137 downregulation

**Fig. 4:**
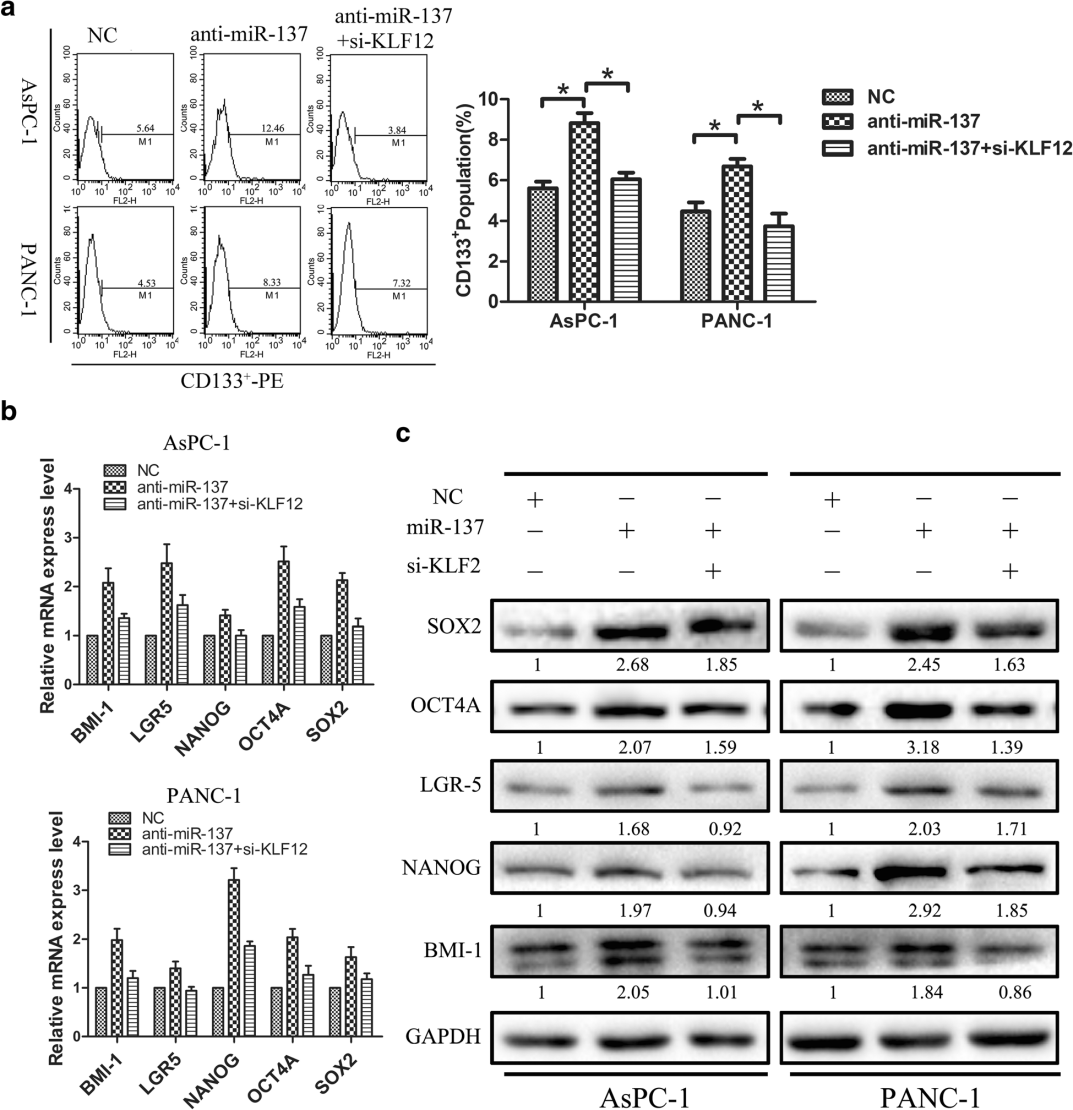
KLF12 mediates CSC phenotype induction after miR-137 downregulation. **a** Silencing KLF12 reduced CD133^+^ populations after downregulation of miR-137. **b** Real-time PCR analyses showed that downregulation of KLF12 inhibited the expression of pluripotency-associated markers in AsPC-1 and PANC-1 cells. **c** Western blot analyses showed that downregulation of KLF12 inhibited the expression of pluripotency-associated markers in AsPC-1 and PANC-1 cells

Next, we investigated whether KLF12 activity mediates miR-137-dependent CSC marker expression in in AsPC-1 and PANC-1 cells by silencing KLF12 gene expression in cells with downregulated expression of miR-137. As shown in Fig. 4a, KLF12 silencing significantly reversed CD133^+^ population increases induced by miR-137-downregulation. In addition, real-time PCR and western-blot analyses showed that the expression of the pluripotency-associated markers BMI1, NANOG, LGR5, OCT4A, and SOX2 was inhibited in these cells in Fig. 4b-c. Thus, our overall results suggest that miR-137 and KLF12 effectively interact to suppress CSC formation and proliferation in human pancreatic cancer cells.

### Suppression of *KLF12* by miR-137 inhibits Wnt/β-catenin signaling

**Fig. 5:**
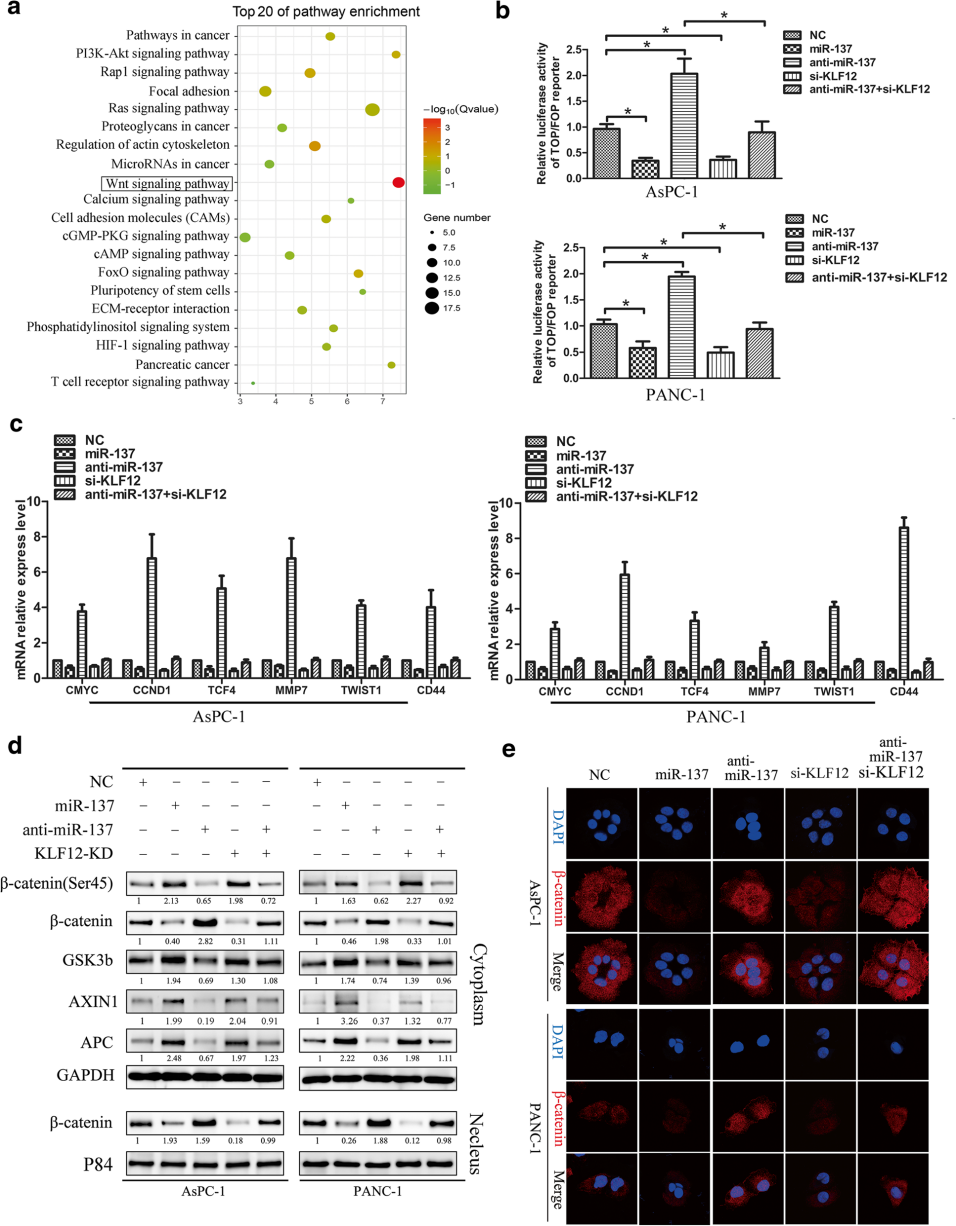
Suppression of *KLF12* activity by miR-137 prevents Wnt/β-catenin signaling in pancreatic cancer cells. **a** KEGG pathway analysis showing that KLF12 expression was positively correlated with Wnt-activated gene signatures in the TCGA pancreatic cancer data set. **b** The indicated cells were transfected with TOP or FOP reporter and Renilla pRL-TK plasmids and subjected to dual-luciferase assays 48 h after transfection. The detected reporter activity was normalized to the Renilla activity. **c** RT–qPCR analysis of the expression of the established downstream targets for the Wnt/β-catenin pathway, including TCF4, TWIST, CMYC, MMP7, CCND1 and CD44 in the indicated cells **d** Western blot analyses the expression levels of AXIN1, APC, GSK-3B, β-catenin, phosphorylation β-catenin on Ser45 and nuclear β-catenin in the indicated cells **e** Subcellular β-catenin localization in the indicated cells was assessed by immunofluorescence staining

In order to study the molecular biological mechanism involving miR-137, and target gene KLF12 regulating pancreatic cancer cell stemness. By performing KEGG-pathway analysis in the TCGA Pancreatic adenocarcinoma data set, we found that the KLF12 level was positively correlated with Wnt-activated gene signatures (Fig. 5a), suggesting that KLF12 might be involved in Wnt/β-catenin signaling activation. Subsequently, we transfected PANC-1 and AsPC-1 cells with miR-137-control,miR-137-mimic, miR-137-inhibitor, si-KLF12, co-transfection with miR-137-inhibitor and si-KLF12 respectively, to further examine the effects of miR-137 and KLF12 on the Wnt/β-catenin pathway. As shown in Fig. 5b,c over-expression of miR-137 in PANC-1 and AsPC-1 cells significantly decreased the activity of the luciferase reporter driven by Wnt/β-catenin signals and the expression of several well-established downstream target genes of the Wnt/b-catenin pathway, whereas the transactivating activity of β-catenin was markedly increased in response to miR-137 inhibiting. Silencing KLF12 produced the consistent with miR-137-mimic,and down-regulation of KLF12 can partially inhibit the function of miR-137-inhibitor effect. Immunohistochemistry was used to detect the association between KLF12 and β-catenin expression in the subcutaneous implanted tumor. The results of immunohistochemistry indicated that the expression of KLF12 and β-catenin was higher in the control group, while the expression of KLF12 and β-catenin was lower in the miR-137 up-regulated group in the Additional file 1: Figure S1. As shown in Fig. 5d, miR-137-mimic significantly promoted AXIN1 and APC, GSK-3β, the phosphorylation of β-catenin on Ser45 expression, and reduced the β-catenin of cytoplasm and nuclear expression in pancreatic cancer cells. Meanwhile, the immunofluorescence staining assays showed that nuclear β-catenin expression decreased significantly in miR-137-mimic and si-KLF12 cell (Fig.5e). whereas decreased in miR-137-inhibitor cells, this effect of miR-137 inhibitor was significantly attenuated by KLF12 knockdown. These results demonstrate that miR-137 represses KLF12-mediated Wnt/β-catenin signaling activation in pancreatic cancer cell lines, and highlight a potential mechanism for miR-137-mediated suppression of stemness properties in pancreatic tumors.

### KLF12 transcriptionally regulates DVL2–β-catenin signaling in pancreatic cancer cells

**Fig. 6:**
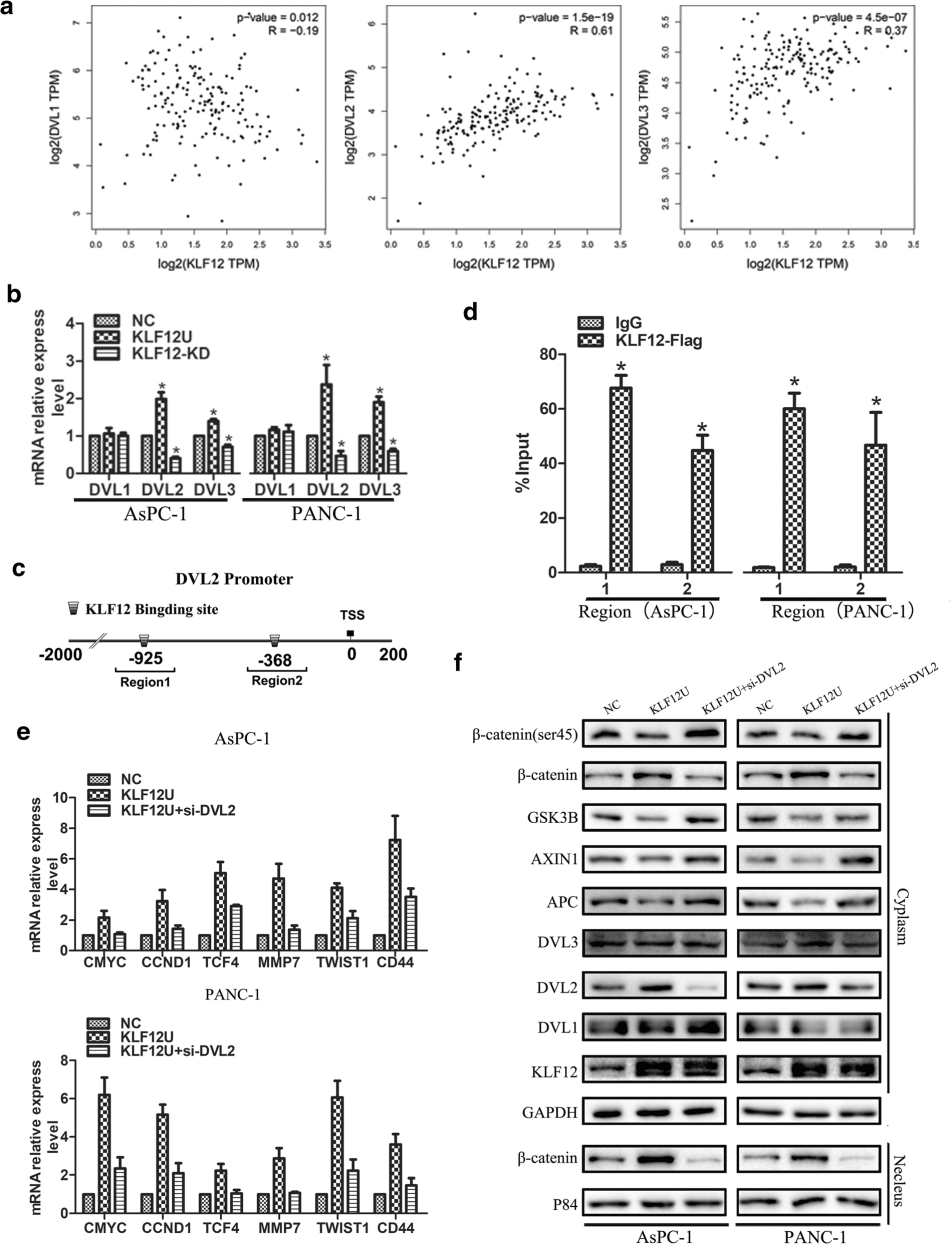
KLF12 transcriptionally regulates DVL2–β-catenin signaling in pancreatic cancer cells. **a** TCGA analysis showing that the expression correlation of KLF12 and DVL1,DVL2,DVL3 in the TCGA pancreatic cancer data set. **b** RT–qPCR analysis of the expression of DVL1,DVL2,DVL3 in the indicated cells **c** Bioinformatics analysis showing that the DVL2 promoter contains two putative KLF12 DNA-binding motifs . **d** Chromatin Immunoprecipitation assays indicated that KLF12 may bind to the promoter regions of DVL2.**e** RT–qPCR analysis of the expression of the established downstream targets for the Wnt/β-catenin pathway in the indicated cells. **e** The expression of Wnt-activated /suppressed gene and nuclear β-catenin in the indicated cells, as determined by western blotting

We next used bioinformatics analysis to identify genes that are regulated by KLF12 Additional file 2. The cytoplasmic protein Dishevelled (DVL1,DVL2,DVL3), which protects β-catenin from a protein complex containing APC, GSK3b, AXIN1. Therefore, we first examined which above candidates have positive correlation with KLF12 expression using analysis of TCGA, showing that level of DVL2,DVL3 associated significantly and positively with KLF12 expression (Fig. 6a). We verified the above results by q-PCR and the results were consistent with the above analysis, after overexpression of KLF12, there was no significant difference in the expression of DVL1, and the expression of DVL2 and DVL3 was significantly up-regulated (Fig. 6b). KLF12 has been reported to bind to the CACCC motif of target genes to regulate their expression [24]. Then, we examined which above candidates have CACCC motif in DVL2 and DVL3 promoter regions. Interestingly, the DVL2 promoter contains two putative KLF12 DNA-binding motifs (CACCC) located at -925 bp (Region 1) and -368 bp (Region 2) relative to the transcription start site (see Supplementary Material 2,3:DVL2 promoter, DVL3 promoter). The results indicated that KLF12 may bind to the promoter regions of DVL2. This was confirmed by chromatin immunoprecipitation assays (Fig. 6c,d). Our results demonstrate Additional file 3 that KLF12 transcriptionally regulates DVL2. Furthermore, small-interfering RNA (siRNA) for DVL2 (si-DVL2) inhibited the effect of increased KLF12 activity on downstream target genes levels in this pathway (Fig.6e), whereas the cytoplasm and nucleus β-catenin was markedly decreased (Fig. 6f).

### KLF12 expression correlates with DVL2 and canonical Wnt pathway in clinical pancreatic cancer

**Fig. 7:**
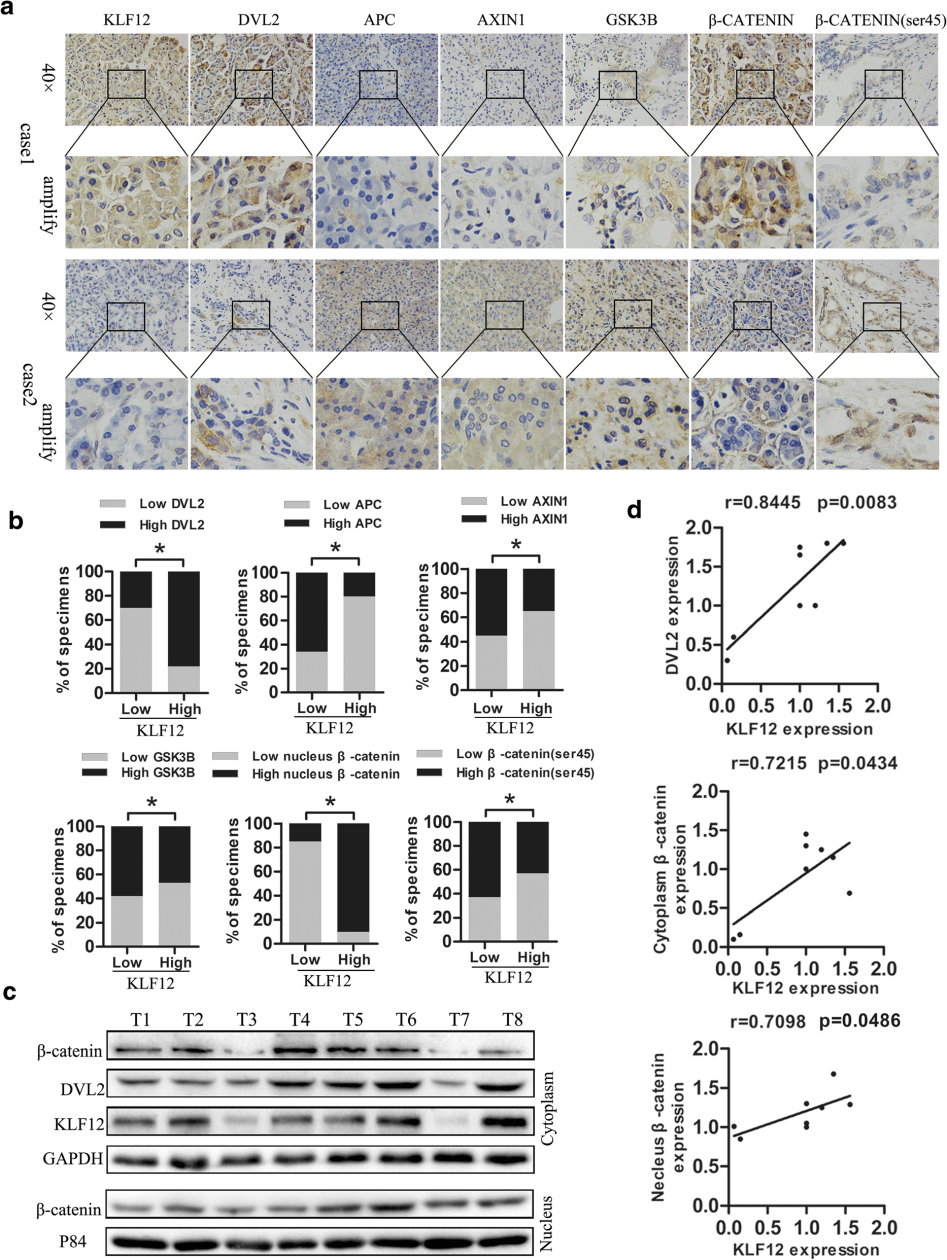
KLF12 expression correlates with DVL2 and canonical Wnt pathway in clinical pancreatic cancer. **a**. Immunohistochemical analysis of KLF12,DVL2, AXIN1, APC,GSK3B,β-catenin, the phosphorylation of β-catenin on Ser45 expression in serial sections of pancreatic cancer specimens.b.KLF12 level correlates with canonical Wnt pathway in clinical pancreatic cancer specimens. **c**-**d**. Eight freshly collected glioma specimens were subject to western blot, showing the correlation of KLF12 expression with DVL2, cytoplasm and nuclear β-catenin

Next, we investigate the clinical relevance between KLF12 level and canonical Wnt pathway in human pancreatic cancer specimens. KLF12 level was correlated positively and significantly with DVL2 expression (*p* < 0.05), nuclear β-catenin expression (*p* < 0.05), demonstrated by IHC (*n* = 24; Fig. 7a, b). Eight freshly collected clinical pancreatic cancer specimens were examined using analysis of western blot, showing that level of KLF12 associated significantly and positively with DVL2 expression (r = 0.8445, *p* = 0.0083), cytoplasm β-catenin expression (r = 0.7098, *p* = 0.0434), as well as nuclear β-catenin expression (r = 0.7098, *p* = 0.0486) (Fig. 7c,d). Taken together, our findings further supported the notion that elevated KLF12 expression activates canonical Wnt pathway through transcriptional regulating Dvl2, which subsequently prevents β-catenin degradation and promotes β-catenin translocated to the nucleus in pancreatic cancer.

## Discussion

Pancreatic cancer continues to be a highly lethal malignancy, despite the use of multimodal treatment approaches [25]. The CSCs statement has been proposed to explain the high rate of relapse and subsequent resistance of cancer to current systemic treatments [26]. Although CSCs constitute a small subset of the total tumor cell population, they are thought to be responsible not only for tumorigenesis, metastasis, and recurrence, but also for tumor heterogeneity and drug resistance [27]. These features reveal that CSCs are involved in the development of tumors, indicating that molecular and pathological characterization of CSCs is important to improve the prognoses of cancer patients. CSCs have been identified in a variety of solid tumors, including pancreatic cancer [28]. However, the precise roles and association between CSCs and the prognoses of patients are accurately understood, especially in pancreatic cancer.

MicroRNAs (miRNAs) exert widespread gene regulation, affecting the expression of oncogenes and tumor suppressor genes that impact CSCs features and modulate tumor growth and resistance to therapy [29]. Therefore, elucidating the underlying mechanism of miRNAs in tumor development may provide valuable diagnostic and therapeutic strategies for malignancy. Several miRNAs, which are involved in stemness maintenance and differentiation, have been identified to play a critical role in regulating pancreatic cancer tumorigenesis signaling networks [30]. Therefore, targeting pancreatic CSCs and elucidating the underlying mechanisms of miRNA in pancreatic CSCs may improve diagnostic and therapeutic strategies for pancreatic cancer.

The miRNA-137 was previously identified to be epigenetically silenced in various types of cancer and to play important roles in tumor development and progression [21–23, 31, 32]. However, the clinical significance of the miRNA-137 family in pancreatic cancer, and the molecular mechanisms underlying the deactivation of its target genes in tumorigenesis and drug resistance derived by pancreatic CSCs still require elucidation.

As our previous work showed that miR-137 levels affect growth, invasion, and sensitivity to chemotherapy in pancreatic tumors [27], we carried out the present study to test the hypothesis that miR-137 is a stemness regulator of human pancreatic cancer cells. To uncover the underlying mechanism, three target gene prediction websites were used to forecast target genes of miRNA-137. KLF12, one of the target genes with a higher predictive score, has been shown to be regulated by miR-137 in gastric cancer [32]. And the potential candidate Krüppel-like factor (KLF) family member KLF12, which was regarded as a transcriptional factor, was selected and monitored in PANC-1 and AsPC-1 cells with knocked down and overexpressed miRNA-137. In this context, the mRNA and protein expression of KLF12 was markedly inhibited by miRNA-137. Owing to the typical seed sequence in the 3′-UTR of KLF12, a direct role of miRNA-137 was naturally surveyed next. As expected, KLF12 was a direct downstream target of miRNA-137, as demonstrated by luciferase assays. The members of the KLF family function as transcription factors and are widely expressed in a number of tissues and organs, they have been reported to control the expression of genes and possess many regulatory functions related to cellular proliferation, differentiation, anoikis, the cell cycle, and cell apoptosis [33–35]. In order to explore the function and downstream signal pathways of KLF12, combining with the TCGA pancreatic cancer data set analysis, our results revealed that KLF12 was significantly correlated with activation of the Wnt/β-catenin signaling pathway. Aberrant Wnt signaling activation has been identified in many solid tumors [36]. Notably, the expression or nuclear localization of β-catenin is often abnormal in cancer cell, indicating a constitutive activation of Wnt/β-catenin signaling [37]. However, how abnormal upstream signal molecule change in wnt/β-catenin signaling cascade, leading to constitutively activated Wnt/β-catenin remains unclear. Herein, we demonstrated that KLF12 as the target of miR-137 induced activation of wnt/β-catenin promotes the stemess of pancreatic cancer cells. To explore the impact of miR-137 and KLF12 on Wnt/β-catenin signaling pathway, we tested the activity of Wnt/β-catenin signaling pathway by performing TOP/FOP Luciferase assays. The activity of Wnt/β-catenin signaling and expression of downstream molecules was significantly decreased in miR-137-overexpress and KLF12-downregulated pancreatic cancer cells, but was increased in miR-137-inhibited cells. Therefore, our findings reveal a novel mechanism for activation of wnt/β-catenin pathway involving miR-137/KLF12 in pancreatic cancer.

When canonical Wnt activated, the Wnt receptors phosphorylate the cytoplasmic protein Dishevelled (Dvl), which protect β-catenin from ubiquitination degradation [38]. Upregulation of Dvl sustains Wnt/β-catenin signaling, whereas Dvl2 loss suppresses tumor development in the intestine of the APC-mutant mouse, suggesting Dvl2 may represent a potential target for colorectal cancer therapy [39]. DVL2 has been consider as an important intermediates of Wnt signaling pathways that inhibits GSK3B,APC,AXIN complex formation. DVL2 is highly expressed and plays an important role in the β-catenin mediated TCF-dependent transcriptional activity, promoting the proliferation of NSCLC cells [40]. Interestingly, We found that the expression of KLF12 was found to be significantly correlated with DVL2 through the TCGA database. Further, we found that KLF12 could transcriptional regulate the expression of DVL2 by q-PCR and Chip experiments. Therefore, we have reason to speculate that KLF12 may transcriptionally activate DVL2 and promote the sustained activation of WNT signaling pathway. Finally, we found a significant correlation between KLF12 and DVL2, β-catenin expression in clinical samples, further supporting the above hypothesis.

Accordingly, we found that miR-137 inhibits the expression and nuclear translocation of β-catenin, and this effect can be partially suppressed by KLF12. Therefore, we propose that miR-137 inhibits pancreatic cancer stemness properties and tumorigenicity by targeting KLF12 via preventing nuclear translocation of β-catenin and activation of Wnt signaling. Further exploration of the upstream molecular events modulating the miR-137/KLF12 interaction, and the resulting impact on Wnt/β-catenin signaling-mediated CSC induction, might shed new light on pancreatic cancer development and progression.

## Conclusion

**Fig. 8:**
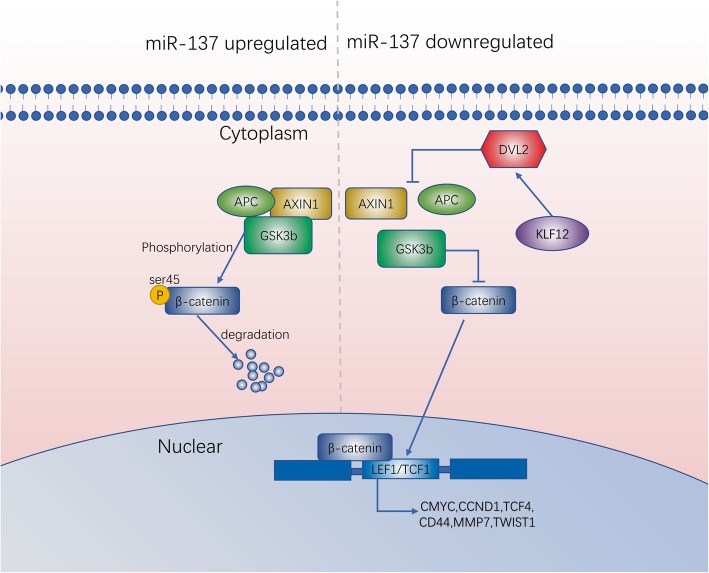
Illustration of suppression of KLF12 activity by miR-137 prevents Wnt/β-catenin signaling in pancreatic cancer cells

In summary, we have identified for the first time that the microRNA-137 reduces stemness features of pancreatic cancer cells by targeting KLF12 inhibit Wnt/β-catenin signaling pathway (Fig.8).

## Additional file

Additional file 1: **Figure S1.** Immunohistochemistry was used to detect the association between KLF12 and β-catenin expression in the subcutaneous implanted tumor. (TIF 4341 kb)

Additional file 2: The sequence of DVL2 promoter. (DOCX 16 kb)

Additional file 3: The sequence of DVL3 promoter. (DOCX 16 kb)

## Abbreviations

ChIP: Chromatin immunoprecipitation
CSC: Cancer stem cell
IHC: Immunohistochemical
MiRNAs: MicroRNAs
qRT-PCR: Quantitative real time polymerase chain reaction
TCGA: The Cancer Genome Atlas
