# Supplementary figures and images for: MicroRNA-137 reduces stemness features of pancreatic cancer cells by targeting KLF12
Source: J Exp Clin Cancer Res. 2019 Mar 12;38:126. doi: 10.1186/s13046-019-1105-3 (PMC6416947; doi:10.1186/s13046-019-1105-3)

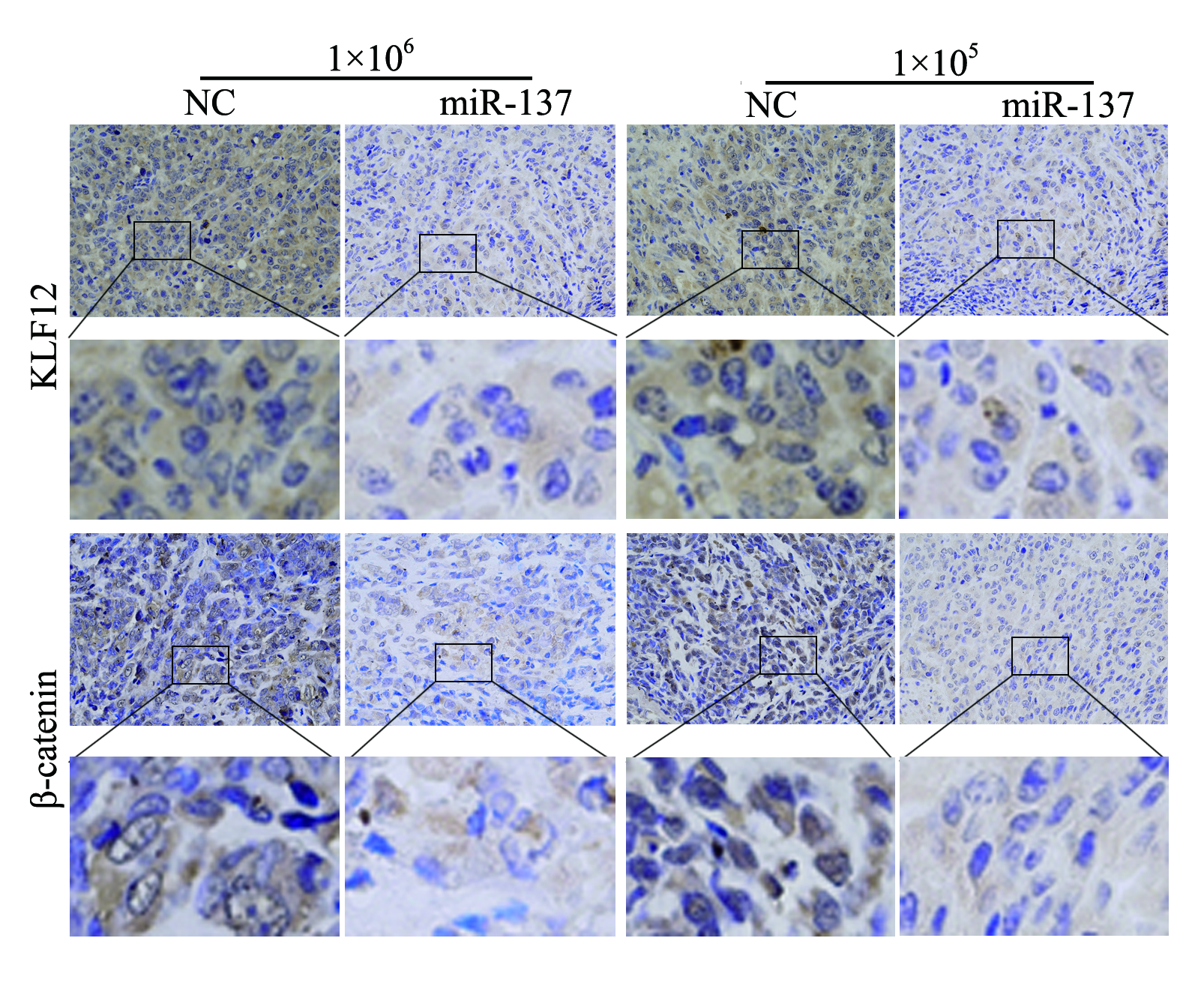

Supplement: Supplementary file 1 — Figure S1. Immunohistochemistry was used to detect the association between KLF12 and β-catenin expression in the subcutaneous implanted tumor. (TIF 4341 kb) [file 13046_2019_1105_MOESM1_ESM.tif]
