# Supplementary material for: MicroRNA-137 reduces stemness features of pancreatic cancer cells by targeting KLF12
Source: J Exp Clin Cancer Res. 2019 Mar 12;38:126. doi: 10.1186/s13046-019-1105-3 (PMC6416947; doi:10.1186/s13046-019-1105-3)
Supplement: Supplementary file 2 — The sequence of DVL2 promoter. (DOCX 16 kb) [file 13046_2019_1105_MOESM2_ESM.docx]

GTTCGGGCTGGGGCGAGGGGTTTGAGGGTGTTGTGGATCCAACAGCTCAGGCAGAATACC

AAACAAACCTTTTTTTCCCCAGGTGATGACTCATGGGATCTGATCACATGTTACTGTCGA

AAGCCCTTTGCAGGGCGGCCCATGATTGAGTGCAGCCTGTGTGGGACGTGGATCCACCTC

TCCTGTGCTAAGATTAAGAAGACCAACGTCCCCGACTTCTTTTATTGCCAGAAATGCAAG

GAACTGAGGCCAGAGGCCCGGCGGTTAGGGGGGCCTCCCAAATCTGGAGAGCCCTGATGG

CACCAACTTTAGCCTGGAACTTCCAAATGACAACATGATTTGGGAACTGAGCCTCAGGGT

CCTCAGCCTATCCCCTGGAGCTTGGATACTGTCTGCACTTCAAGGCAGGAATTCTCAAGG

GAGACTTGTTTGAAAATGAcGTGTCTCACTTTCCCACCCTATCCTTCCTCCCCACTCTGTG

GACTTGAAATTGAATCCATTACGGTTGGGGATGGGAGGCTGTCTGTGTCCCGACACATAA

TCTCTGTCTCTTGGACCTGCCACCATCACTTTCTGGGTCAGGATTGGAATTGGGATGGAA

TGGGACAGTTGTCTATAAAACTCTAGTGTAAATATTAGCACTCCCCTCCCTCATCTTTTC

TTCTATTTCACTCCCCATTTATTTTCTTCTACACCGGTTGTATTTTTAATTTTGGACTTC

CCCTATTGGGCATGGCAGCTCAAAGGTGGAGTACTAGAGCCTGGCCAAGTGAGGAAGGAA

AGCAGAAAGGTGACGATTCTCACTCACCTCTTTTGTTTTTAATAATATCGGCCGCTGTTT

GTACAGACAGCCTGCGTGTTGTAAATAAAGCAGAGTGGGCTCTTTTGTGTTTATAGCCCT

CACTGTCCTGCTCGGGAGGGAGGCTTTGGTGGCTGAAGACTAGCTGGAGGTGGGGCTGAA

ACCCACGTCTCAAAGTCCAATCTGAAGATATCACAGCACTGGGCCTTTCCCACCCCAAGA

CAAGTAAGCCACCTTAGAGCTTTCATTGTATTTGGCCCTCCCCACTCGAGTCTAGGAACC

CCCACAGTCCCCACCTTCACTCCCTGCACGCACACTCCTGCGGGTCAGAGTTGCTTCGTG

CTCCATCGTGGGTGGGGTAAAGCGGGGTGGGAGCCGATGGTCCGTTTCCTGAAGGCACAG

GCCAGCTTAGCCTGGGTGCTCAGAGGACCTCGGCTGGGGTACACGTTTGGGGGCTGGGAG

ACCGTTGCTGAAGGCAGCGATCATGTGGCGGGGGCACTAGGCGGAGTCAGACGGTCACGT

GATTGGGAGGGGATCACGTGACGGAGGGCGGCTGAGGAGCGCGGGGTGGCTGCCTTTA**A**G

TCACGTGACATGAGGAGAGGTGGGCGGGTACCTGGAGGAAGCTCGCGGCGTCGGTGGCGG

TGGCGCGCGGCGGCCGCTGAGACCGGGGCTTTGAGTCGCACCCCGCGGCCCGCCCCCCGC

CGCCACCCTCGCAGATCCGTGCTTTTTCCCCTTTGCTTCTCTCCCGTACTGGGTCAGTCC

TGTCCGCGCTCGCGCGTC
