# Supplementary material for: MicroRNA-137 reduces stemness features of pancreatic cancer cells by targeting KLF12
Source: J Exp Clin Cancer Res. 2019 Mar 12;38:126. doi: 10.1186/s13046-019-1105-3 (PMC6416947; doi:10.1186/s13046-019-1105-3)
Supplement: Supplementary file 3 — The sequence of DVL3 promoter. (DOCX 16 kb) [file 13046_2019_1105_MOESM3_ESM.docx]

AGCCATTGCGCCTGGCTTCCCCTGTCACTATCCCATCACTTTGTTTTACCTTCTTCATAG

CAGTGGTCACTCTGAAGTTATGTATTTACAAGCCTATGTGCTTCCCTCCAGCTGAGGGAA

GGTCCTGAGAGCAGCCTTGTCTGTCTCGCTATCGCTATATCTCATGCGATATGCCTTATA

CACAGTAGGCCTTCAGTATTTATTAAATGAATTAAAAGTTAAGGGGTGAGGAGATCTGTG

AGGAACCGGCTATACCTAGTTCTGGAGTTCTGGTTTGAGACTTACTGTCTTAGGCATGCA

TGCATCCACTTATCTTTTTTTTTGGGGGGGGGCGAGGGGGGATGGAGTTTCACTTTTGTT

GCCCAGGCTGGAGTGCAAAGGCAGGATCTCGACTCACCGATCTCGGCTCACCGCAACCTC

CGCCGCCCGTGTTCAAGCGATTCTTCTGCCTCAGCCTCCCGAGTAGCTGAGATTACATGC

ATGCGCCACCACGCCCGGCTAATTTTTTTGTATTTTTAGTAGAGACAGGGTTTCTCCATG

TTGGTCAGGCTGGTCTTGAACTCTCGACCTCGGGCGATCCGCCCGCCTTGGCCTCCCAAA

GTGCTGGGATTAGAAGCGTGAGCCACCGCGCCCGGGCCCCACTTATTCCGCATTCAACCC

ATTTATTAAACCACTACCTTGCAACTGGACGCTAAGGTTATAGGGAGGAATCAGAACTGG

ATGCTGTCCTTCTCTCGAGATCAGTTTAGTAGACTCAGTGACAGCAGTTGTGCTTCTCAA

GAAACACTGAAGGGCCATGTTGAGGTCATTCTCCTCCAGGGATCCAGAGTCAGACCCACC

CTCTTTCCCCAGACCATTTCTGGGGACCAAAGCAATCCCTGCATTCCACTCTTCCTGGAC

TGCAGAGGAGTCGCCGATCCCACTTGGGTCCGGAGTCAGGGCCTTCAGGGGTCCGGGAAC

GCCGCCATCCCGGTGAATCTCCCCTTCCGTTTCTAGGGACCCGGGACCGGAGCGCCGCGG

CCCGGCCAGTGGGCGGGAAACCCTGCCATCTGCCGCGACGCCGGCGCCTCTCCGCGGCGG

CCGGGGGCGGGGCGCGCCGGGGCGGGGCCGCGCCGGGCCGGAGGAGGGGAGCGGGCCCGG

ACGAAGCCCTGGGCCGGGAGGGCCGCGGCCACCGGAAGAGTCGCGGTCGCCAGTCCAGTC

GGGAGAGTGGGGAGCGGAAGCGGCGGCCGCGGCGGCGGCGGGCGGCGCTGGGACCCGGTA

GCGGCCGGA**G**AACAAGGGAGCTGGCGCCGCCAGCAGCCGCCGAGCTGGGTTGAGCCGCT
